# Age-dependent pathogenic profiles of enterotoxigenic *Escherichia coli* diarrhea in Bangladesh

**DOI:** 10.3389/fpubh.2024.1484162

**Published:** 2024-12-12

**Authors:** Marjahan Akhtar, Yasmin Ara Begum, Sadia Isfat Ara Rahman, Mokibul Hassan Afrad, Nasrin Parvin, Afroza Akter, Imam Tauheed, Mohammad Ashraful Amin, Edward T. Ryan, Ashraful Islam Khan, Fahima Chowdhury, Taufiqur Rahman Bhuiyan, Firdausi Qadri

**Affiliations:** ^1^Infectious Diseases Division, International Centre for Diarrhoeal Disease Research, Bangladesh (icddr,b), Dhaka, Bangladesh; ^2^Division of Infectious Diseases, Massachusetts General Hospital, Boston, MA, United States; ^3^Department of Medicine, Harvard Medical School, Boston, MA, United States; ^4^Department of Immunology and Infectious Diseases, Harvard T.H. Chan School of Public Health, Boston, MA, United States

**Keywords:** ETEC, diarrhea, age, toxins, colonization factors (CFs), AMR

## Abstract

**Background:**

Age plays a significant role in susceptibility to enterotoxigenic *Escherichia coli* (ETEC) infections, yet the distribution of ETEC virulence factors across age groups remains understudied. This study investigated the differential pathogenic profiles ETEC across various age groups, emphasizing the importance of selecting potential ETEC antigens tailored to infection patterns in infants and adults in Bangladesh.

**Methods:**

This study utilized the icddr,b’s 2% systematic hospital surveillance data of diarrheal patients (*n* = 14,515) from 2017 to 2022 to examine the age-specific pathogenesis and clinical manifestations of ETEC infections.

**Results:**

In total ETEC was identified in 1,371 (9.4%) of surveillance samples. ETEC-associated diarrhea was higher in children aged 0–2 years and decreased significantly in the 3–17 years age group. Among all ETEC cases, 56% were adults (*p* = 0.0079) with severe dehydration. Distinct age-specific distribution of ETEC toxin types and colonization factors (CFs) were observed: heat labile toxin (LT)-associated ETEC infections decreased with age (*p* < 0.0001), while heat stable toxin (ST)-associated-ETEC was prevalent across all ages. Adults exhibited significantly higher rates of ETEC diarrhea with strains secreting both types of toxins. A high prevalence of antimicrobial resistance among ETEC strains, particularly in pediatric cases, with significant resistance observed against commonly used antibiotics such as azithromycin and in line with similar age specific toxin profiles. The most common CFs were CFA/I, CS3, CS5, CS6, and CS21. CFA/I positive ETEC infection was more common in children (*p* < 0.001), while CS5 and CS6 were more common in adults (*p* < 0.0001).

**Conclusion:**

The findings provide valuable insights into ETEC epidemiology, pathogenesis, and clinical manifestations. These observations imply that age-related differences in host-pathogen interactions exist for ETEC infections and this may influence the development of targeted vaccines or therapeutics and use in specific populations.

## 1 Introduction

Enterotoxigenic *Escherichia coli* (ETEC) is a major cause of bacterial diarrhea in children. Annually, ETEC imposes a staggering burden, contributing to approximately 220 million reported cases and causing 380,000 fatalities (1, 2), mostly in low and middle-income countries (LMICs) with inadequate sanitation and hygiene practices. Furthermore, ETEC infection significantly contributes to childhood stunting and growth faltering as well as lifelong disabilities (1, 3). ETEC-induced diarrhea is also common in travelers to endemic areas (4). Infection is established when ETEC reaches the intestinal mucosa, and expresses a number of fimbriae that bind specific cellular targets. These fimbriae are collectively referred to as colonization factors (CFs) that enable ETEC attachment to the mucosal epithelium. Notably, there are over 30 distinct CFs and adhesion antigens identified in ETEC strains globally (5). After attachment and colonization, the bacteria proliferate and produce heat-labile enterotoxin (LT) and/or heat-stable enterotoxin (ST) on the epithelial surface which disrupt normal intestinal function, leading to watery diarrhea (2, 6–8). However, the pathogenesis of ETEC involves complex interactions between the bacterium, immune responses as well as gut microbial features of the host (9).

Enterotoxigenic *Escherichia coli* vaccine development is mainly based on targeting most prevalent CFs as well as the LT toxin because of their strong immunogenic properties. Phase I/II clinical trials of a second generation ETEC vaccine, ETVAX, consisting of four recombinant *E. coli* strains overexpressing CFA/I, CS3, CS5 and CS6, and a CTB/LTB hybrid protein (LCTB*A*), has been carried out for safety and immunogenicity in non-endemic and endemic countries including Bangladesh (10–15). The vaccine was found to be safe and strongly immunogenic in Bangladeshi children and adults (11, 12). However, antibody responses to LT and CFs antigens were observed in non-vaccines (placebo recipients), suggesting high level endemic exposure (11, 12). Among the placebo recipients, immune responses against different ETVAX CFs antigens varied in different age groups indicating potential age-specific preferences in *CF* responses. For instance, higher levels of mucosal and systemic antibody responses against all vaccine CFs and LT antigens were found in 6–11 month old infants compared to older age groups and adults suggesting high levels of exposure in early age (12). In addition, phase I trial of this ETVAX vaccine in Zambian children aged 10–23 months also reported high placebo responses (14). The higher antibody responses among non-vaccines suggest early and high exposure to ETEC in endemic areas in different age groups.

Enterotoxigenic *Escherichia coli* infection induces diarrhea across all age groups, yet infection rates vary among different demographic regions with a predominance among younger individuals (2, 16). From icddr,b 2% hospital surveillance data and other studies in Bangladesh, it is evident that age plays a significant role in susceptibility of ETEC infection (2, 17–19). However, the distribution of ETEC toxin and CFs across age groups remains understudied. In this investigation we aimed to elucidate the differential patterns of toxin and *CF*-associated ETEC across various age cohorts, emphasizing the importance of selecting potential ETEC antigens with robust immunogenic properties tailored to the infection patterns observed in infants and adults. Specifically, we aimed to analyze age-specific differences in the pathogenicity of ETEC infection, focusing on the burden of ETEC toxin and CFs in different age groups in Bangladeshi participants.

## 2 Materials and methods

### 2.1 Study design

This study used the demographic data from the 2% systematic surveillance system of icddr,b where diarrheal stool specimens are collected from every 50th patient presenting for clinical care. Microbiologic, demographic, disease severity, clinical management, and disease outcome of each surveillance patient is recorded. In this study, diarrheal stool specimens (*n* = 14,515) collected from patients from 2017 to 2022 (we were not able to include 2020 data due to interrupted enrollment during the initial stage of the COVID-19 pandemic). Stools were screened to determine *Vibrio cholerae*, ETEC, Salmonella and Shigella spp. ETEC positive diarrheal cases (*n* = 1,371) from the study period was studied to evaluate the burden and distribution of ETEC infection associated with different toxins and CFs in different age groups. Demographic information of these patients are shown in Table 1. The 2% systematic surveillance of icddr,b is an ongoing study that has been approved by the Research Review Committee (RRC) and Ethical Review Committee (ERC) of icddr,b ‘Institutional Review Board (IRB). Verbal consent was taken from patients or from the guardians or caregivers of the patients following hospitalization, for permission to use the data for research and publication.

**Table 1:** Demographics of enterotoxigenic *Escherichia coli* (ETEC) diarrheal patients.

Parameters	Patients, *n* (%)
ETEC diarrhea	1,371
Sex	
Male	757 (55%)
Female	614 (45%)
Age distribution	
0–1 year	300 (22%)
>1–2 years	162 (12%)
>2–5 years	76 (6%)
>5–17 years	61 (4%)
≥18 years	772 (56%)

### 2.2 Determination of ETEC toxins and colonization factors

For detection of ETEC, stool specimens were streaked on MacConkey agar and then incubated overnight at 37°C. A multiplex PCR method was used to determine the presence of ETEC toxin LT and ST as described previously (2, 20). Next, the toxin positive colonies were plated onto colonization factor antigen (CFA) agar with and without bile salts and was tested for expression of CFs; CFA/I, CS1, CS2, CS3, CS4, CS5, CS6, CS7, CS8, CS12, CS14, and CS17 using *CF*-specific monoclonal antibodies by dot-blot immunoassay (21). Isolates were also cultured on Trypticase Soy Agar (TSA) plates and tested for CS21 as previously described (17, 18).

### 2.3 Antimicrobial susceptibility test

Antibiogram assay was performed to determine the antimicrobial susceptibility of ETEC strains against commonly used antibiotics using the guidelines of National Committee for Clinical Laboratory Standards as described previously (22). The antibiotic disks used in the study included ampicillin (10 μg), azithromycin (15 μg), ceftriaxone (30 μg), ciprofloxacin (5 μg), doxycycline (30 μg), erythromycin (15 μg), mecillinam (25 μg), nalidixic acid (30 μg), norfloxacin (10 μg), streptomycin (10 μg), sulfamethoxazole-trimethoprim (25 μg) and tetracycline (30 μg). *E. coli* ATCC 25922, susceptible to all antimicrobials was used as a control strain for susceptibility studies.

### 2.4 Statistical analysis

Descriptive statistics for all the demographic and clinical characteristics are presented as frequencies and percentages. Pearson’s Chi-square test was used to measure associations with factors contributing to ETEC infection in different age group of patients. All statistical tests have been conducted at 5% level of significance. Data were analyzed by using Excel, GraphPad Prism (version 6.0).

## 3 Results

### 3.1 Frequency of ETEC diarrhea in different age groups

**Figure 1:**
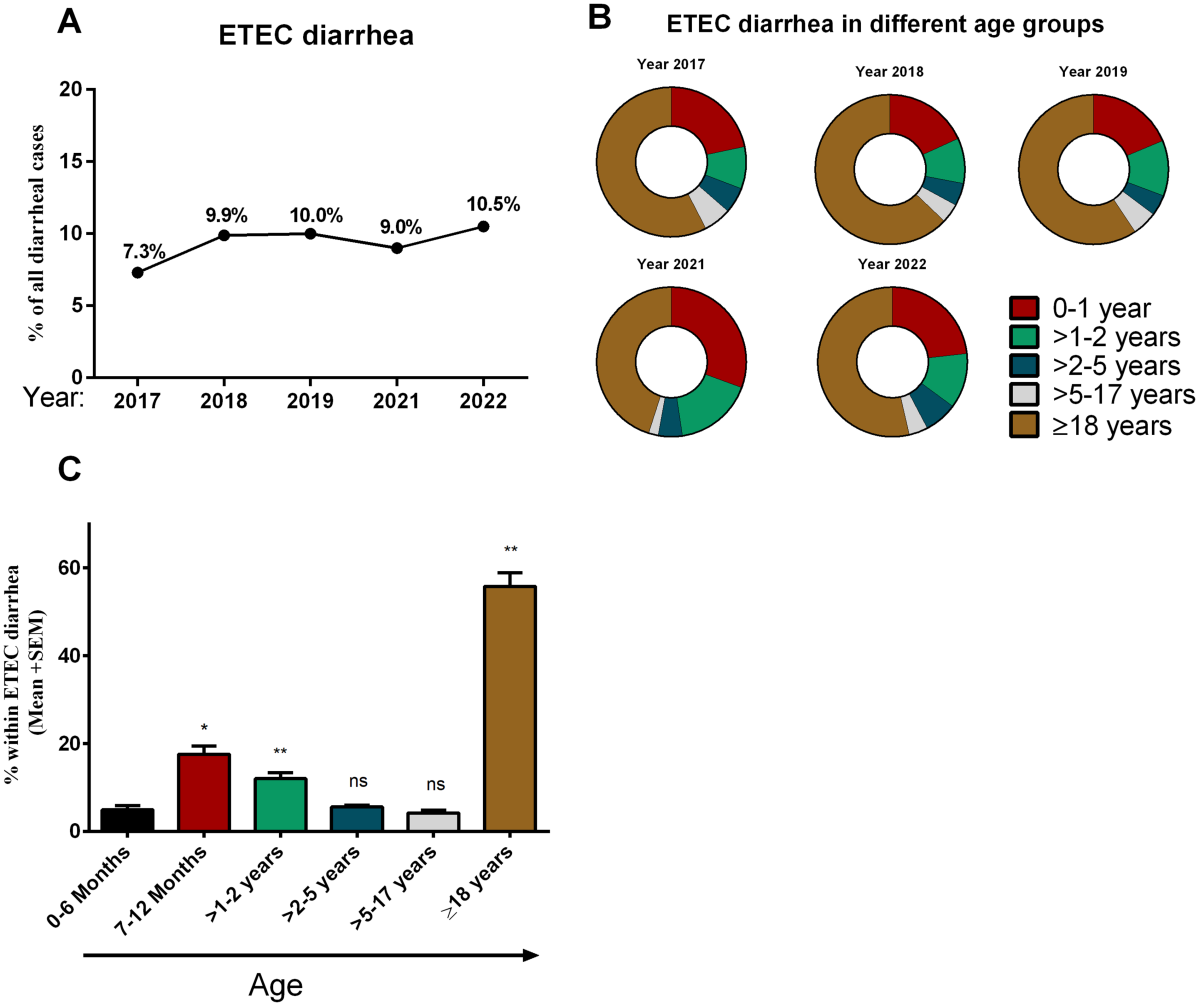
Enterotoxigenic *Escherichia coli* (ETEC) diarrhea and its distribution in different age groups. **(A)** Rate of ETEC diarrhea in different years (2017–2022) among diarrheal patients, **(B)** distribution of ETEC diarrhea in different age groups in different years and **(C)** comparison between the rate of ETEC diarrhea in different age groups. Bars indicate Mean + SEM of the percentage of ETEC in any age group among total ETEC cases in five different years. Statistical analysis was performed between 0 and 6 months versus other age groups using the Mann–Whitney *U* test. ^*^p < 0.05, ^**^p < 0.01, ^***^p < 0.001; ns, not significant; *p* > 0.05.

We tested 14,515 hospitalized patients with diarrheal symptoms during the study period. Among these, 1,371 (9.4%) tested positive for ETEC antigens. ETEC cases were 7.4% in 2017 and gradually increased in the following years to 10.5% in 2022 (Figure 1A). We evaluated the distribution of ETEC-associated diarrhea across different age groups (Figures 1B,C). Approximately, 5% (67 of 1,371) of ETEC cases occurred within the first 6 months after birth, then increased significantly until 24 months of age (17–22%). Following 2 years of age, there was a relatively low frequency (4–6%) of ETEC cases observed in a broad age range spanning from children aged 25 months (>2 years) to 17 years old. Among all age groups, adults (>18 years) had the highest rate of ETEC infection (56%) during the study period. Co-infection with *Vibrio cholerae* O1 was detected in 24% ETEC infected diarrheal cases, with the majority of these co-infections (18%) were in adults.

### 3.2 Pathogenic profile of ETEC expressing different toxins in different age groups

**Figure 2:**
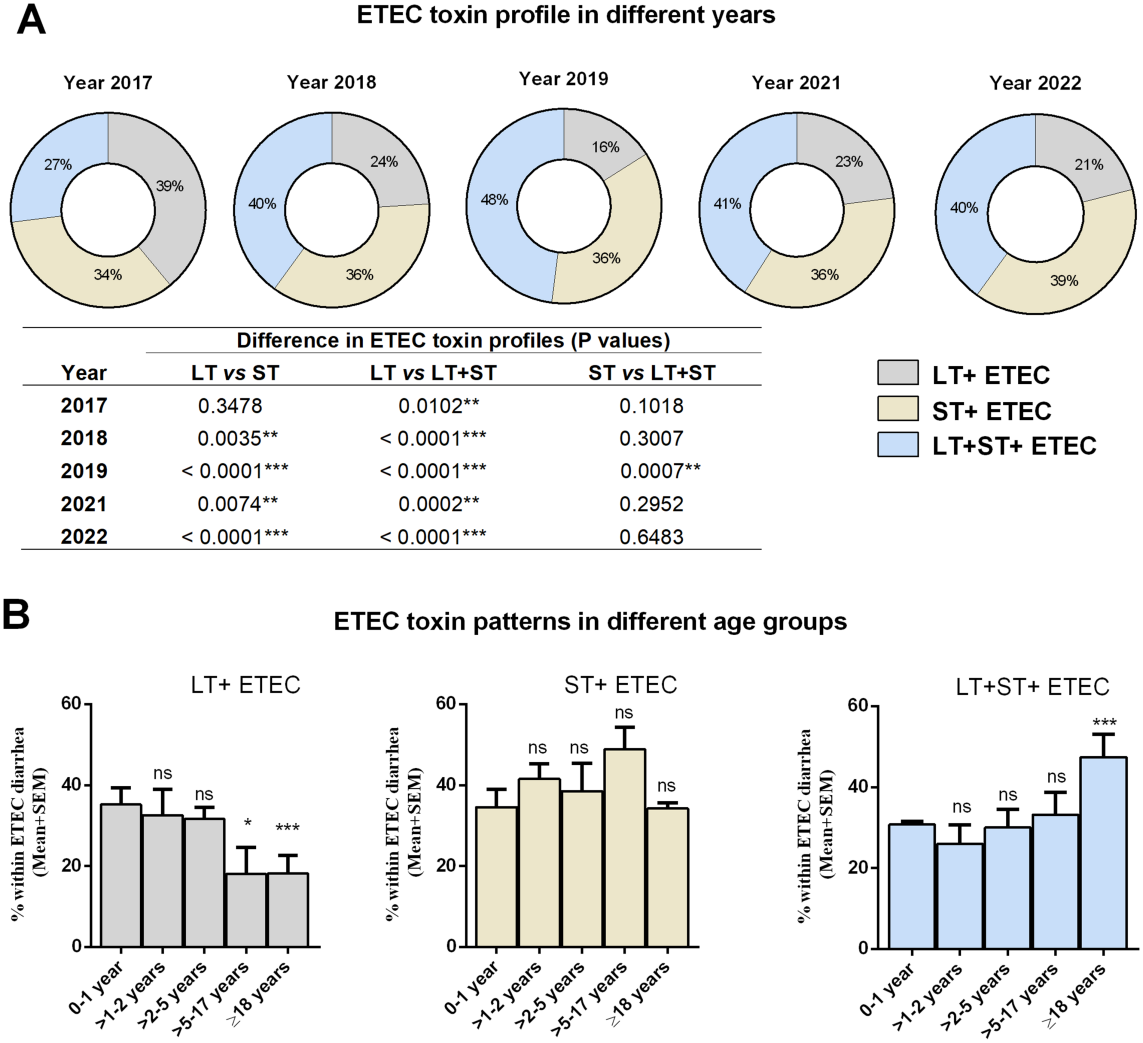
Distribution of ETEC enterotoxins in different years and age groups. **(A)** ETEC toxin (LT, ST, and LT + ST) profiles in different years. Statistical analysis was performed between LT vs. ST, LT vs. LT + ST and ST vs. LT + ST using the Chi-square test. **(B)** ETEC toxin (LT, ST, and LT + ST) profiles in different age groups. Statistical analysis was performed between 0 and 1 years versus other age groups using the Chi-square test. ^*^p < 0.05, ^**^p < 0.01, ^***^p < 0.001; ns, not significant; *p* > 0.05.

First, we evaluated the prevalence of ETEC enterotoxins LT and ST across different years (Figure 2A). In 2017, LT (39%) was the predominating toxin, followed by ST (34%) and LT + ST (27%), among the clinical ETEC isolates. However, there was a shift in enterotoxin patterns in the subsequent years. From 2018 onwards, both LT + ST (40%) expressing ETEC isolates along with ST expressing (36%) increased. By 2022, diarrhea caused by ETEC strains expressing ST alone became more prevalent compared to LT alone (21%) (Figure 2A).

Next, we were interested in assessing whether ETEC strains expressing LT, ST, or both toxins displayed distinct association in causing diarrhea in different age groups (Figure 2B). Within the 1 year old age group, nearly all toxin patterns were equally prevalent. The prevalence of LT-positive ETEC infection decreased with increasing age. Adults (>18 years) had a significantly lower rate of LT+ ETEC infection (17%) compared to youngest age groups (34%). There was no significant difference in ST+ ETEC infection across age groups. However, ETEC strains expressing both LT + ST toxins showed a preponderance in adults (48%) compared to the younger age groups (31%) (Figure 2B).

### 3.3 Colonization factors profile of ETEC strains

**Figure 3:**
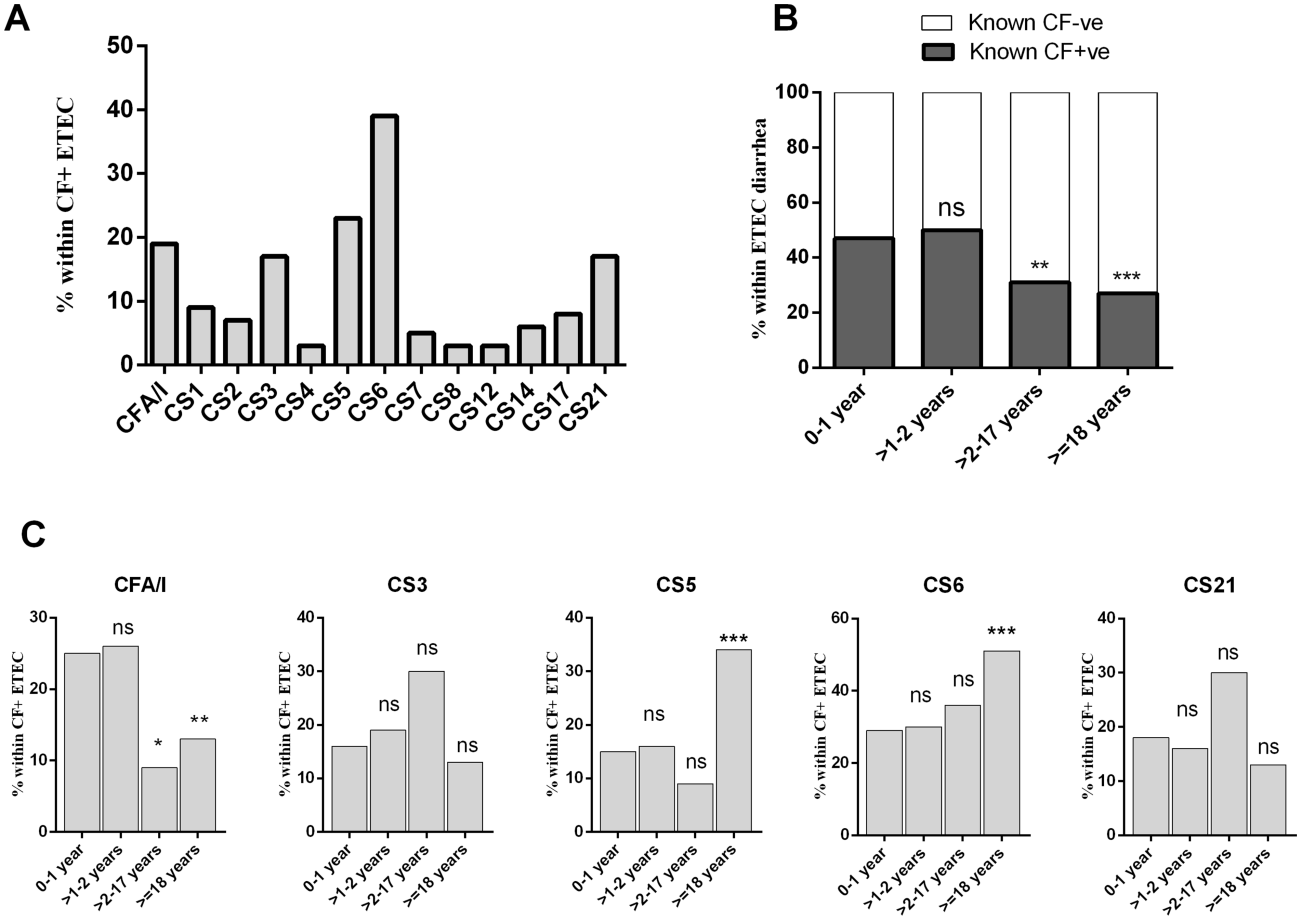
Enterotoxigenic *Escherichia coli* expressing different colonization factors (CFs) isolated from diarrheal patients. **(A)** Frequency of ETEC expressing 13 different colonization factors, **(B)** frequency of *CF* + ve and *CF*−ve ETEC across diarrheal age group patients and **(C)** distribution of prevalent CFs (CFA/I, CS3, CS5, CS6, and CS21) in different age group. Bars represent the frequency of CFs among all ETEC cases. Statistical analysis was performed between 0 and 1 years versus other age groups using the Chi-square test. ^*^p < 0.05, ^**^p < 0.01, ^***^p < 0.001; ns, not significant; *p* > 0.05.

We determined 13 distinct ETEC CFs using a dot blot assay in a total of 1,371 ETEC strains isolated from hospitalized diarrheal patients. ETEC strains that tested positive for any of the more common 13 CFs were categorized as “known *CF*+” type. Among cases of ETEC-positive diarrheal illness, 35% (*n* = 476) were associated with known *CF* + ve ETEC infection. During the five-year study period, ETEC expressing CS6 (39%), CS5 (23%), CFA/I (19%), CS3 (17%), and CS21 (17%) either alone or co-expressed with other CFs were the most prevalent CFs among the “known *CF*+” clinical strains (Figure 3A).

In terms of age-specific *CF*+ ETEC infection, the highest rate (47%) of *CF* + ETEC causing diarrhea were observed in the >2 years age group. In comparison to younger age groups, older children (>2–17 years, 20%, *p* < 0.001) and adults (>18 years, 27%, *p* < 0.0001) had significantly lower rates of known *CF*+ ETEC-associated diarrhea (Figure 3B). Furthermore, we evaluated the frequency of the most prevalent CFs in different age groups (Figure 3C). Interestingly, distinct frequencies of CFA/I, CS5 and CS6 expressing ETEC infection were observed across age groups. The frequency of CFA/I+ ETEC diarrhea decreased with age, with 0–2 years children exhibiting significantly frequent CFA/I associated ETEC diarrhea compared to older children and adults. Conversely, CS5+ or CS6+ ETEC diarrhea increased with age, with adults showing the highest rate of CS6 (53%, *p* < 0001) and CS5 (37%, *p* < 0001) associated ETEC diarrhea compared to younger age groups (Figure 3C). On the other hand, CS3+ ETEC diarrhea was present across all age groups.

### 3.4 Association of ETEC toxins and CFs

**Figure 4:**
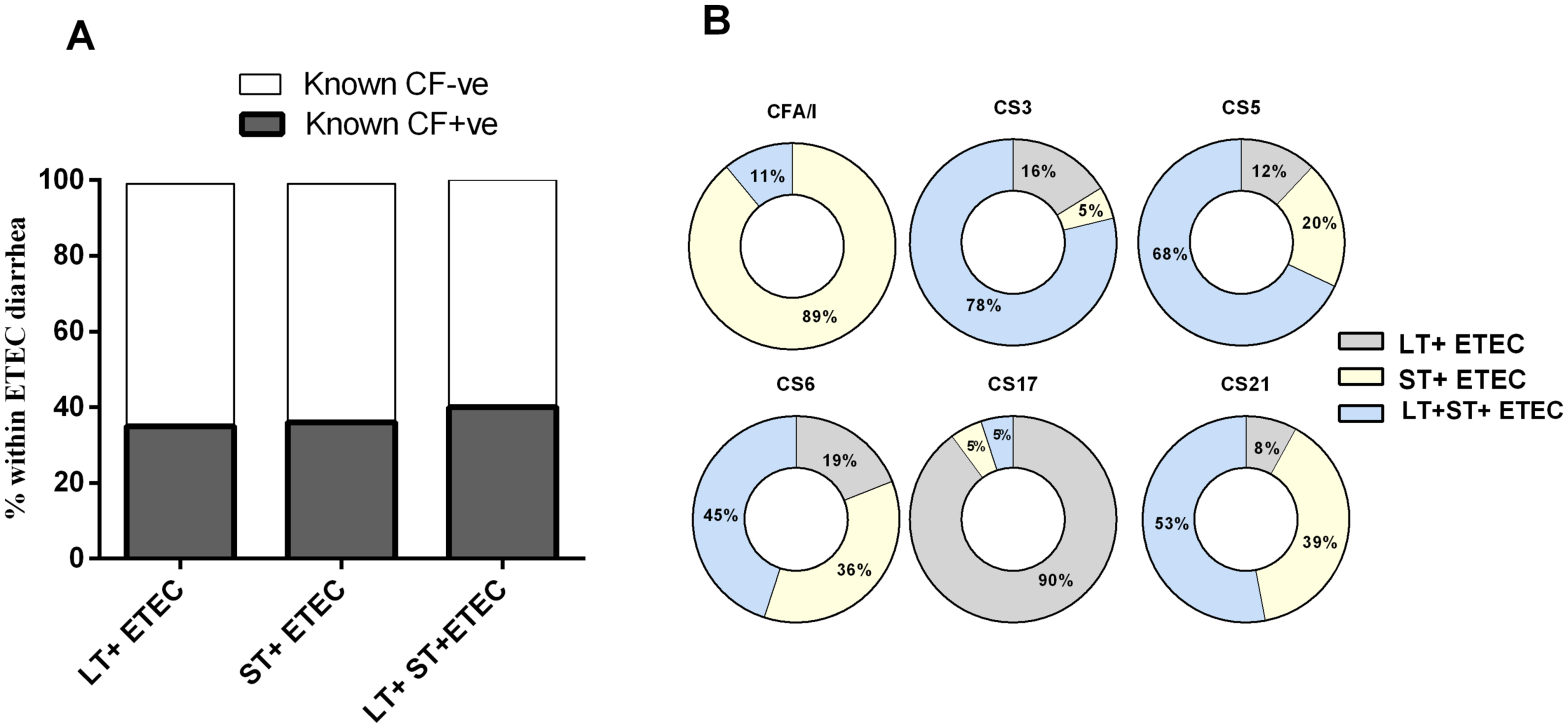
Enterotoxigenic *Escherichia coli* (ETEC) colonization factors (CFs) and its relation to toxins. **(A)** Toxin profiles of CFs positive and negative ETEC and **(B)** Association of prevalent CFs (CFA/I, CS3, CS5, CS6, CS17 and CS21) with carrying three different toxin types.

Given that ETEC can express two distinct enterotoxins- LT and ST- in three different combinations (LT, ST or LT + ST), alongside alone or co-expression of >30 different CFs, there may be a correlation between the expression of specific CFs and the presence of particular toxins within ETEC strains. Nearly an equal percentage of either toxin-positive ETEC strains express known CFs (Figure 4A). Our analysis focusing on major CFs revealed that CFA/I (89%) was of the most common ST+ ETEC strains. ETEC strains harboring both LT and ST toxins carried mostly CS3 (78%) and CS5 (68%) and CS6 (45%). CS6 was found to be predominantly present on LT + ST (45%), followed by ST+ (36%) and LT+ (19%) ETEC strains. The majority of CS17 (90%) was linked with LT+ ETEC strains. Additionally, CS21 was identified on LT + ST (53%), ST (39%) and LT+ (8%) ETEC strains (Figure 4B).

### 3.5 Diarrheal dehydration status in different age groups

**Figure 5:**
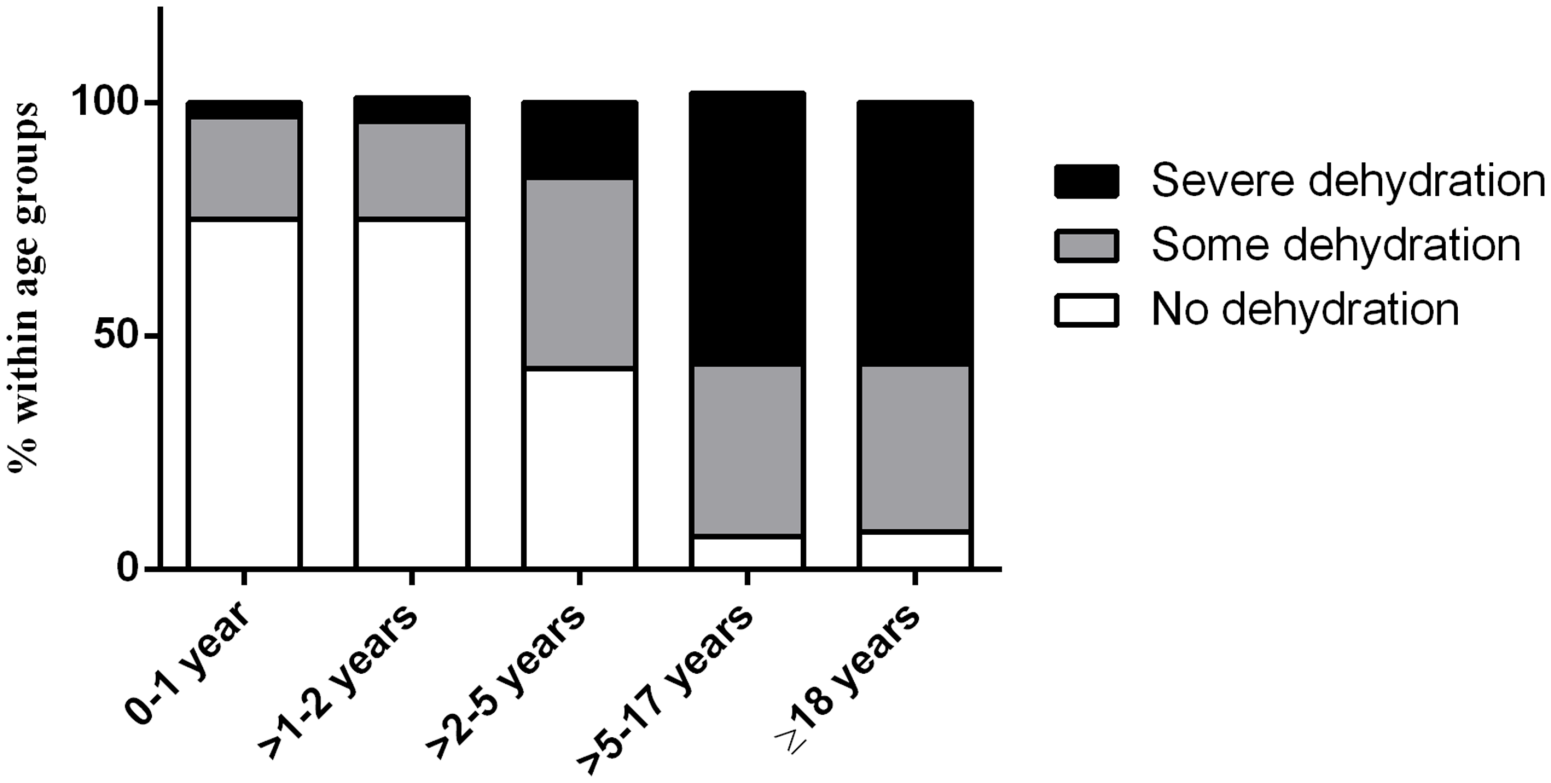
Dehydration status of ETEC diarrheal patients in different age groups.

We evaluated the dehydration status, according to WHO guidelines, in hospitalized ETEC-infected diarrheal patients (*n* = 1,038) across various age groups, after excluding those with a history of *Vibrio cholerae* co-infection. Our analysis revealed a correlation between diarrheal severity and age (Figure 5). Among children aged 0–2 years (*n* = 434), 75–76% showed no signs of dehydration, while 20% experienced mild dehydration and only 3–5% experienced severe dehydration. In the >2–5 years age group (*n* = 57), 49% had no signs of dehydration, 37% had mild, and 14% experienced dehydration. Alternately, among patients age > 5 years, including adults (*n* = 547), over half (56%) experienced severe dehydration, 36% had mild dehydration and only 7–8% showed no signs of dehydration during the onset of diarrhea symptoms (Figure 5).

### 3.6 Antimicrobial resistance (AMR) pattern in ETEC

**Figure 6:**
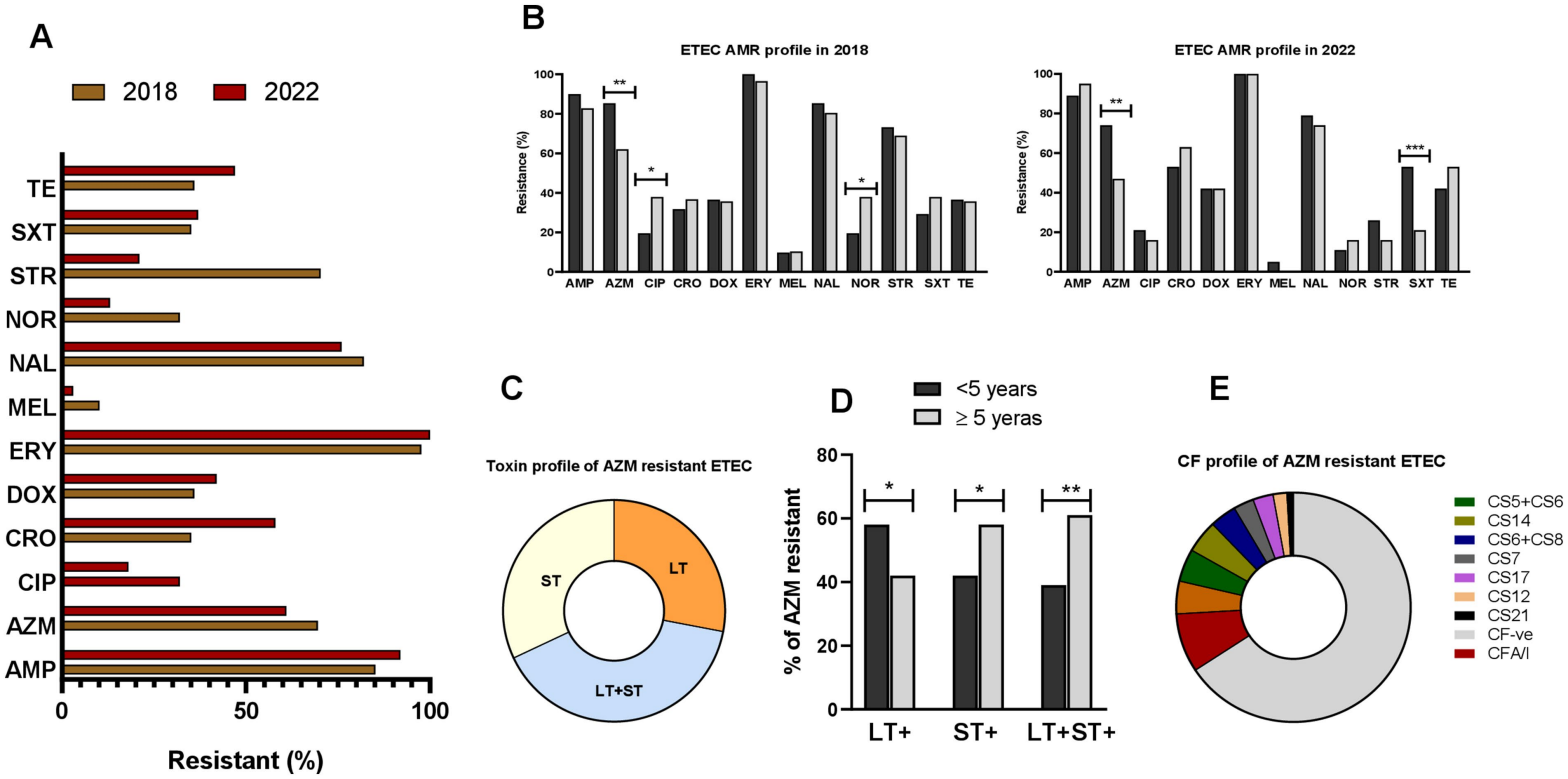
Antimicrobial resistant (AMR) profile of ETEC. **(A)** Resistance profile of ETEC strains to ampicillin (AMP), azithromycin (AZM), ciprofloxacin (CIP), ceftriaxone (CRO), doxycycline (DOX), erythromycin (ERY), mecillinam (MEL), nalidixic acid (NAL), norfloxacin (NOR), streptomycin (STR), sulfamethoxazole-trimethoprim (SXT) and tetracycline (TE), isolated from diarrheal patients in 2018 and 2022. **(B)** Comparison of ETEC AMR resistance profile in <5 years and ≥5 years old diarrheal patients. **(C)** Toxins profile of azithromycin resistant ETEC strains. **(D)** Differences in toxin profiles of azithromycin resistant ETEC isolated from <5 years and ≥5 years old diarrheal patients and **(E)** colonization factors profile of azithromycin resistant ETEC strains. Statistical analysis was performed between <5 years and ≥5 years using the Chi-square test. ^*^p < 0.05, ^**^p < 0.01, ^***^p < 0.001.

We analyzed AMR in a subset of ETEC strains (*n* = 166) isolated from diarrheal patients in 2018 and 2022. Most ETEC strains showed high resistance to the 12 tested antibiotics, with over 50% showing resistance to ampicillin, azithromycin, ceftriaxone, erythromycin, nalidixic acid, and tetracycline (Figure 6A). Age specific differences in AMR pattern were also observed. Between age groups (<5 years vs. ≥5 years), significantly resistant differences were observed for azithromycin, ciprofloxacin and norfloxacin in 2018 and for azithromycin and sulfamethoxazole-trimethoprim in 2022 (Figure 6B). ETEC strains from children diarrhea showed significantly higher (*p* < 0.001) azithromycin-resistance compared to ETEC strains from adult cases. Based on this, we assessed toxin profiles of the azithromycin-resistant ETEC strains; among them 27% LT+, 32% ST+ and 39% LT + ST+ ETEC were found (Figure 6C). Next, we evaluated the toxin profiles of azithromycin-resistant ETEC in children and adults. Notably, children showed higher (*p* < 0.05) azithromycin resistant LT + ETEC infection compared to adults. In contrast, adults had higher azithromycin resistant LT + ST+ ETEC and ST+ ETEC infections (*p* < 0.001 and *p* < 0.05, respectively) compared to children (Figure 6D). Within azithromycin resistant ETEC strains, a majority of the ETEC strains were known *CF* negative, while CFA/I (8.3%), CS2 + CS3 + CS21 (4.6%), CS5 + CS6 (4.6%) and CS14 (4.6%) were common among the *CF*+ ETEC strains (Figure 6E). Interestingly, no CFA/I+ was found within the azithromycin sensitive ETEC strains (data not shown).

## 4 Discussion

The study utilized the icddr,b’s 2% systematic surveillance in diarrheal patients from Dhaka city over a multiyear period. The findings of this study shed light on several key aspects of ETEC infection, including its age-specific pathogenic profile and clinical manifestations. Understanding these dynamics is crucial for informing targeted vaccine design to reduce the burden of ETEC-associated diarrheal disease. One important aspect of our study is the observed variation in the frequency of ETEC diarrhea across different age groups. This finding underscores the importance of considering ETEC as a significant cause of diarrheal illness in both pediatric and adult populations. At the very early phase following birth up to 6 months, a relatively low prevalence of ETEC infection was observed among hospitalized diarrheal patients, which notably increased during the first 2 years of life. This trend is also similar as shown earlier by our group that ETEC associated diarrhea is reported mostly in <5 years age children (17–19). Nonetheless, our analysis revealed that most of the ETEC cases within 5 years of age were concentrated in children less than 2 years old, and children aged more than 2 years, ETEC-associated diarrhea became infrequent. Our data highlights the vulnerability of young children (0–2 years old) to ETEC infection, likely due to factors such as immature immune systems and environmental exposures. Consistent with earlier studies (18), another peak of ETEC infections was observed among adults (>18 years), comprising the highest proportion (56% of all ETEC diarrhea) of ETEC infections during the study period.

Our findings suggest distinct frequencies for specific ETEC toxins among different age groups. LT-ETEC infection decreased with increasing age, perhaps reflecting protection afforded by previous exposure to this immunogenic antigen. 34% of the children less than 1 year had LT-ETEC associated diarrhea while only 17% adults had LT-ETEC associated diarrhea. Our findings also indicate a high prevalence of antimicrobial resistance among ETEC strains, particularly in pediatric cases, with significant resistance observed against commonly used antibiotics such as azithromycin. Additionally, age-specific differences in toxin profiles of azithromycin resistant ETEC infection also in line with observed infection dynamics, with children more susceptible to LT + ETEC, while adults exhibit higher rates of LT + ST+ ETEC infections. Since LT is super immunogenic (23–26), children infected with LT-ETEC may induce protective immunity to prevent LT-associated diarrhea in the older life. In contrast, ST-ETEC was commonly found in all age groups of diarrheal patients including children. This can be also explained by the non-immunogenic properties of ST (7); ST-ETEC associated diarrhea in children is probably unable to induce immunity and might be responsible for the increased rate of ST-ETEC associated diarrhea in all age groups. In a birth cohort study conducted during 2007–2009 in Mirpur, Dhaka, Bangladesh, also showed the prevalence of ST-ETEC diarrhea in children (17). Another study conducted in Peru also reported that high proportion of ST-ETEC infection in children (16). The rate of ETEC secreting both toxins LT + ST is significantly higher in adults compared to children. To our knowledge, no study has done prevalence of ETEC toxin associated diarrhea in different age groups. One of the limitations of this study is that we only studied symptomatic diarrheal patients who visited the hospital, but no data are available for asymptomatic ETEC infection. Moreover, our analysis also revealed shifts in the prevalence of ETEC toxins over time, with strains expressing ST alone becoming increasingly prevalent compared to LT alone. This shift in toxin expression patterns may reflect evolving pathogenic mechanisms within ETEC populations and could have implications for disease severity and clinical outcomes.

Enterotoxigenic *Escherichia coli* expresses a variety of colonization factors that are also very important components in candidate ETEC vaccines (27, 28). Most of the vaccine design is based on the most prevalent CFs like CFA/I, CS3, CS5 and CS6 (11, 27, 29, 30). However, along with these prevalent CFs, our analysis showed CS21 (17%) as a commonly associated *CF* in diarrheal patients of all age groups. The Peruvian birth cohort children also shown prevalence of CS21 expressing ETEC infection in both symptomatic and asymptomatic cases. However, in the Peruvian cohort CS12 expressing ETEC was also predominant (13.2%) (16). CS12-expressing ETEC infection is very minor in Bangladesh, only found in approximately ~3% of all CFs (2, 17, 18). These suggests distinct prevalence and pathogenesis of *CF* associated ETEC in different geographical locations (31). We also observed age-specific patterns in *CF* prevalence, with older children and adults exhibiting lower rates of known *CF*-positive ETEC infections compared to younger age groups. Furthermore, certain CFs showed distinct preferences for infecting specific age groups. CFA/I-associated ETEC diarrhea was more frequent in children aged 0–2 years, whereas CS5 and CS6-associated infections were more prevalent in adults. CFA/I + ETEC strains frequently exhibited resistance to azithromycin in our study, indicating a heightened risk for children. These findings suggest that the distribution of CFs may contribute to age-related variations in ETEC susceptibility and disease presentation.

Most of the ETEC epidemiological studies has been conducted in children (16, 17, 32–36). Our analysis highlights that ETEC is not only a cause of diarrhea in children, it is also significantly associated with adults. About 56% of all ETEC-associated diarrhea occurred in adults aged > 18 years. ETEC infection in adults was associated with severe dehydration. We found that the proportion of patients experiencing severe dehydration increased with age, with adults being particularly susceptible. This observation underscores the importance of prompt recognition and management of dehydration, especially in older patients, to prevent severe complications and improve clinical outcomes. Moreover, vaccination strategy should also target recognizing high ETEC prevalence rates in adult populations in endemic countries such as Bangladesh. Both innate and adaptive immune system of infants or children differs from that of older age groups (37), and antigen selection to make an effective ETEC vaccine should consider these variations. For instance, the Phase I/II clinical trial of the inactivated ETEC vaccine revealed higher levels of mucosal and systemic antibody responses against all vaccine CFs and LT antigens in 6–11 month-old infants compared to older age groups and adults (12). This underscores the importance of tailoring vaccine formulations to optimize immune responses across different age demographics. Notably, CS21, which is pathogenic (38, 39) and found in high frequency in Bangladesh, is not included in most of advanced vaccine candidates (13, 27, 40, 41) except epitope- and structure-based multiepitope-fusion-antigen (MEFA) vaccinology platform (42). Moreover, in addition to ETEC toxin and CFs, vaccine formulation may need to be tailored in adults and pediatric population considering the other novel antigens EtpA and EatA (43). The importance of antigen selection for vaccine development in both infants and adults cannot be overstated, particularly in the context of ETEC vaccine.

Overall, our findings provide valuable insights into the epidemiology, pathogenesis, and clinical manifestations of ETEC infection across different age groups. By elucidating age-specific patterns of toxin expression, *CF* prevalence, and disease severity, our study contributes to the development of targeted prevention and control strategies for ETEC-associated diarrheal disease. Future research efforts should focus on further characterizing the mechanisms underlying age-related differences in ETEC susceptibility and disease progression, with the ultimate goal of reducing the global burden of diarrheal illness caused by this important pathogen.

## Data Availability

The raw data supporting the conclusions of this article will be made available by the authors, without undue reservation.
